# Viral pathogens in children hospitalized with features of central nervous system infection in a malaria-endemic region of Papua New Guinea

**DOI:** 10.1186/s12879-014-0630-0

**Published:** 2014-11-26

**Authors:** Moses Laman, Ilomo Hwaiwhanje, Cathy Bona, Jonathan Warrel, Susan Aipit, David Smith, Joanna Noronha, Peter Siba, Ivo Mueller, Inoni Betuela, Timothy ME Davis, Laurens Manning

**Affiliations:** School of Medicine and Pharmacology, University of Western Australia, Fremantle Hospital, Fremantle, Western Australia Australia; Papua New Guinea Institute of Medical Research, Madang, Papua New Guinea; Modilon General Hospital, Paediatrics department, Madang, Papua New Guinea; PathWest laboratory, School of Pathology and Laboratory Medicine, University of Western Australia, Nedlands, Western Australia Australia; Barcelona Centre for International Health Research, CRESIB, Hospital Clínic-Universitat de Barcelona, Barcelona, Spain; Walter & Eliza Hall Institute, Parkville, Victoria Australia

**Keywords:** Viral encephalitis, Human herpes viruses, Malaria, Meningitis, Dengue

## Abstract

**Background:**

Viral central nervous system (CNS) infections are common in countries where malaria is endemic but, due to limited laboratory facilities, few studies have systematically examined the prevalence and clinical consequences of the presence of viruses in cerebrospinal fluid (CSF) from children with suspected CNS infection.

**Methods:**

We performed a prospective study of Papua New Guinean children hospitalized with signs and symptoms of CNS infection. CSF samples from 300 children without proven bacterial/fungal meningitis were analyzed for human herpes viruses (HHV), picornaviruses, influenza, adenoviruses, flaviviruses and bacteria.

**Results:**

Fifty-five children (18%) had viral (42), bacterial (20) or both viral and bacterial (7) nucleic acids (NA) identified in their CSF. Human herpes viruses accounted for 91% of all viruses found. The identification of viral or bacterial NA was not associated with any characteristic clinical features. By contrast, malaria was associated with increased identification of viral and bacterial NA and with impaired consciousness, multiple convulsions and age. Malaria was also inversely associated with an adverse outcome. Amongst children with HHV infection, those with HHV-6 and −7 were younger, were more likely have impaired consciousness and had a higher proportion of adverse outcomes than children with CMV. Dengue and enteroviral infections were infrequent. Adenoviral and influenza infections were not identified.

**Conclusion:**

Infections with HHV-6, HHV-7, dengue and enterovirus have the potential to cause serious CNS disease in young PNG children. However most HHVs in this malaria-endemic setting should be considered to be the result of reactivation from a latent reservoir without clinical sequelae.

**Electronic supplementary material:**

The online version of this article (doi:10.1186/s12879-014-0630-0) contains supplementary material, which is available to authorized users.

## Background

The diagnosis and management of central nervous system (CNS) infections can be challenging in malaria-endemic tropical countries due to limited laboratory facilities and therapeutic options. In addition, restricted availability of health care services that may also be difficult to access can result in late clinical presentations. Viral pathogens are increasingly identified in patients who present with clinical features of CNS infection in this setting, where they may be i) the principal cause of the illness, ii) an alternative post-mortem diagnosis in patients with an established clinical diagnosis of cerebral malaria [1], iii) co-incident pathogens that influence both the CNS manifestations and subsequent mortality of malaria [2], or iv) as in the case of neurotropic human herpes viruses (HHVs) that can establish a latent reservoir through chromosomal integration of DNA, as a bystander reactivated during an acute illness [3],[4].

The types and proportions of viruses that have been identified in CNS infections vary by geo-epidemiological setting. The CNS symptoms caused by cerebral malaria (CM) are a common indication for lumbar puncture (LP) and reactivation of HHVs in this situation is well described [5]. As a result, geographic variability in the incidence, severity and outcome of CM may impact on the viruses detected in children presenting with possible CNS infections [6]. Other possible factors that might influence viral CNS infection include local variability in entero-, adeno- or arbovirus incidence [7], local zoonotic reservoirs that may facilitate the emergence of novel viral pathogens [8], as well as the choice of virus-specific primers that are included in diagnostic multiplex PCR tests.

Few studies have systematically assessed inter-relationships between the presence of viral nucleic acids (NA) in cerebrospinal fluid (CSF) with specific clinical features and outcome. This is especially important for HHVs such as varicella zoster (VZV), herpes simplex viruses-1 (HSV-1), cytomegalovirus (CMV), HHV-6 and HHV-7 as the presence of NA in the CSF may be result of reactivation of viral NA that have been integrated into host cells and may not necessarily represent a primary acute infection. Such studies are, however, challenging because of the potentially large number of candidate viruses and non-viral comparator groups. In a recent study of 513 hospitalized Malawian children, 12 different viruses were isolated from CSF or post mortem brain biopsies, and the presence of any virus increased the risk of death especially in those children with co-incident malaria [2].

Although febrile encephalopathy is common in malaria-endemic coastal Papua New Guinea (PNG), there have been few etiologic studies. In a study of children diagnosed with likely bacterial CNS infections, no causative organism was found in most cases [9]. We hypothesized, therefore, that viruses contribute significantly to the burden of severe CNS illness and its presentation. As part of a detailed prospective observational study of severe childhood infections, we retrospectively analyzed CSF samples from PNG children hospitalized with signs of a CNS infection but without culture- or latex agglutination-proven bacterial or cryptococcal infections for the presence of i) HHVs, ii) picornaviruses, iii) influenza viruses, iv) adenoviruses, v) flaviviruses and vi) bacterial pathogens (*Streptococcus pneumoniae* (SP)*, Haemophilus influenzae* type b (Hib) *and Neisseria meningitidis*).

## Methods

### Study site

The present study was carried out at Modilon Hospital, the main referral hospital in Madang Province on the north coast of mainland PNG, between November 2007 and July 2010. Severe childhood malaria caused by either *Plasmodium falciparum* and/or *P. vivax* is common [10] and falciparum malaria is known to cause CSF leukocytosis in approximately 21% of children in this setting [11]. Hib and SP are leading causes of acute bacterial meningitis [12], and cryptococcal meningoencephalitis in immunocompetent children occurs occasionally [12]. Hib vaccination was introduced into Madang Province in 2008 and the pneumococcal vaccination was introduced in 2013. Post-measles complications presenting as sub-acute sclerosing panencephalitis (SSPE) have also been relatively common in this area [10]. During the recruitment period of this study, LPs were often performed in children presenting after a single, uncomplicated febrile seizure, in accordance with the PNG standard treatment guidelines at the time [13].

### Clinical procedures

All hospitalized children aged between 2 months and 10 years who presented with febrile seizures, impaired consciousness, coma or other clinical signs and symptoms suggestive of a possible CNS infection were screened for inclusion. A standardized case report form that included demographic and clinical data was completed [14], including details of the presenting illness, characteristics of seizures, vaccination history and past medical history. Trained research nurses and study clinicians carried out clinical assessment on admission. Detailed neurological examinations were performed by study clinicians in patients presenting with coma or neurological features. A Blantyre Coma Score (BCS) ≤4 was considered to represent impaired consciousness [15].

### Laboratory procedures

**Figure 1:**
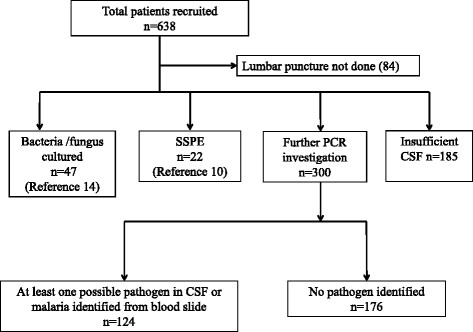
**Study profile outlining investigations performed on cerebrospinal fluid of Papua New Guinean children with possible central nervous system infection.**

Cerebrospinal fluid was collected under sterile conditions and examined using standard microbiological procedures [12]. In brief, an Improved Neubauer Counting Chamber was used to count CSF leukocytes. All CSF with leukocyte counts ≥10 cells/μL were plated onto blood and chocolate agar for bacteriological culture. Cerebrospinal fluid samples with no cultured pathogen underwent latex agglutination testing (Wellcogen™, Remel Europe Limited, UK). Protein and glucose were measured using semi-quantitative dipstick tests. Blood cultures were taken and placed into an automated blood culture incubator (Bactec 9050, BD®). Patients with bacteria or fungus identified from CSF, blood cultures or by latex agglutination were excluded from further analysis (Figure 1).

Cerebrospinal fluid samples were stored at −80°C for further viral and bacterial molecular investigations and subsequently tested in an Australian nationally accredited laboratory. Molecular tests included nested PCR (nPCR) for HHV-6 HHV-7, picornavirus and adenovirus, multiplex real-time PCR for varicella zoster [VZV] and herpes simplex viruses-1 [HSV-1] and −2, tandem multiplex real-time PCR for influenza A and B virus, and for flaviviruses. The presence of CMV, *S. pneumoniae*, *H. influenzae* and *N. meningitidis* was determined using real-time PCR. Quantitative PCRs of viral NA, subtyping of viral species and serological testing was not performed. Malaria in children diagnosed by microscopic examination of thick blood smears was also subsequently confirmed by nPCR [10].

### Ethical approval

Written informed consent was obtained from parent(s)/guardian(s) on admission, and approval for this study was obtained from the PNG Institute of Medical Research Review Board and the Medical Research Advisory Committee of the PNG National Department of Health (MRAC number 10.08).

### Data analysis

Two-way comparisons of proportions were by Fisher’s Exact or Chi-squared tests, and comparisons of medians were by Mann–Whitney *U* and Kruskal-Wallis test for two or more than two groups, respectively. Post-hoc pairwise comparisons were performed using Dunn’s test. Multivariate analysis by logistic regression was performed. Variables with a univariate *P*-values <0.10 were included in a backward stepwise process. The most parsimonious final model was determined using Aikake’s Information Criterion. A two-tailed significance level of *P* < 0.05 was used throughout.

## Results

Of 638 children presenting with clinical features suggestive of CNS infection, a LP was performed in 554 (87%). Of this latter group, definite diagnoses of bacterial/fungal meningitis and SSPE were made in 47 (9%) and 22 (4%) children, respectively. There was insufficient CSF in a further 185 children (33%) and 87 of these had a diagnosis of CM. The remaining 300 (54%) with possible CNS infections had CSF available for molecular testing (Figure 1). The median age of this subgroup was 24 (interquartile range [IQR] 18–29) months and 59% were males. One-hundred and eighty-three children (61%) had convulsions, 47 (16%) were comatose at the time of admission, and 42 (14%) had impaired consciousness.

**Figure 2:**
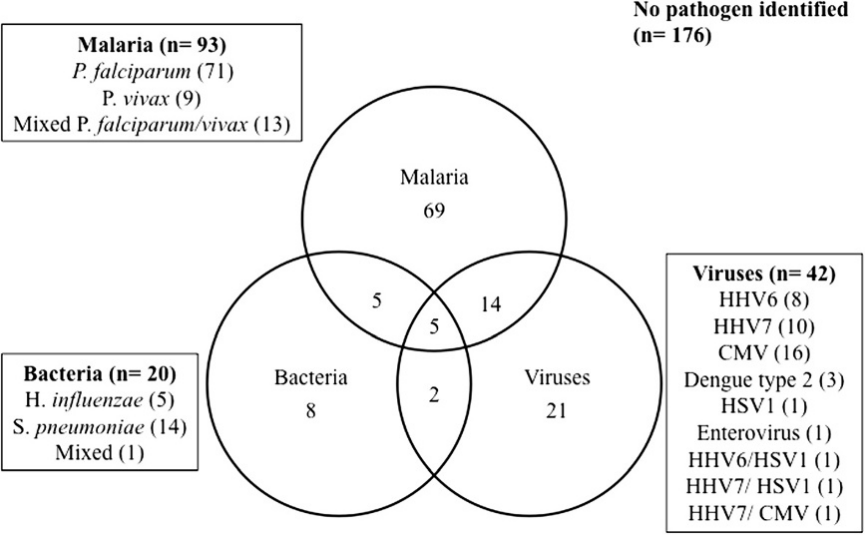
**Overlap between possible viral, bacterial or malarial infection in Papua New Guinean children with possible central nervous system infection.** The identified viral agent or species are shown in the boxes.

Fifty-five of the 300 children (18%) had at least one type of viral or bacterial NA identified in their CSF, of which 42 had at least one virus identified, including 39 and three with single- and dual infections, respectively. Overlap between viral NA, bacterial NA and malaria was common (Figure 2). Bacterial DNA was identified in 20 children, with 7 of these also with viral NA present. Children with bacterial infection included 14, 5 and 1 with SP, HI and both SP/HI, respectively. Malaria was identified in 93 children (31%). An etiologic agent was not identified in 176 children (59%). The characteristics of children by likely etiologic agent according to malaria status are summarized in Table 1. After adjustment for age, the presence of viral and bacterial NA was associated with malaria infection (odds ratio [OR] 2.05 [95% confidence interval (CI_95_)] 1.03-4.08, *P* = 0.04 and 2.62 [1.02-6.8], *P* = 0.04, respectively).

**Table 1:** **Clinical and laboratory characteristics of children in the present study**

	Malaria parasitaemia			No malaria parasitaemia			
Characteristics	Viruses (n = 19)	Bacteria (n = 10)	Malaria alone (n = 69)	Viruses (n = 23)	Bacteria (n = 10)	No pathogen (n = 176)	***P*** -values
Age	40 [29–80]^a^	36 [29–38]^b^	36 [23–60]^c^	7 [5–25]^a^	6.5 [5–18]^b^	15 [6–44]^c^	<0.001
Male sex	11 (57.9)	5 (50)	37 (53.6)	14 (60.9)	5 (50)	110 (62.5)	0.90
Convulsions	13 (68.4)	7 (70)	50 (72.5)	13 (56.5)	2 (20)	102 (58)	0.03
Neck stiffness	1 (5.3)	2 (20)	13 (18.8)	8 (34.8)	4 (40)	37 (21.0)	0.27
Kernig’s sign positive	0 (0)	0 (0)	4 (5.8)	2 (8.7)	0 (0)	9 (5.1)	0.90
Brudzinski’s sign positive	0 (0)	0 (0)	1 (1.4)	1 (4.4)	0 (0)	3 (1.7)	0.96
Impaired consciousness	10 (52.6)	4 (40)	38 (55.1)^c^	5 (21.7)	1 (10)	32 (18.2)^c^	<0.001
Haemoglobin (g/L)	71 [58–106]	78.5 [71–106]	85 [68–102]	91 [65–111]	101.5 [89–106]	96 [84.5–109]	0.07
White blood cells (X10^9^/L)	8.4 [5.7–14.2]	7.4 [5.6–10]	8.6 [5.7–11.7]	7.9 [5.3–15.5]	8.6 [4.8–13.2]	9.8 [7.4–12.7]	0.50
Platelets (X10^9^/L)	100 [63–222]^d^	147 [100–238]	103 [46–206]^c^	368 [197–378]	618 [305–992]	265 [165–390]^c,d^	<0.001
Lactate (mmol/L)	2.7 [2.0–4.6]	1.8 [1.2–2.4]	3.4 [2.0–5.3]	1.2 [1.0–4.2]	4.3 [3.3–15.7]	2.4 [1.9–3.3]	0.01
CSF leukocytes (cells/μL)	0 [0–5]	2.5 [0–30]	0 [0–5]	5 [0–10]	0 [0–5]	0 [0–5]	0.04
CSF protein (g/L)	0.3 [0.3–0.3]	0.3 [0.15–0.3]	0.3 [0.15–0.3]	0.3 [0.3–0.3]	0.3 [0.3–0.3]	0.3 [0.15–0.3]	0.35
Death	1 (5.3)	0 (0)	2 (2.9)	1 (4.4)	1 (10)	9 (5.1)	0.93
Discharged with disability	2 (10.5)	0 (0)	1 (1.4)	2 (8.7)	0 (0)	9 (5.1)	0.46

Data are numbers (percent) for dichotomous variables or medians (interquartile range) for continuous variables. Due to viral and bacterial co-infections in 7 children, numbers do not add to 300.

^a^Significant post-test pairwise comparison (P < 0.05) between malarial and non-malarial infection in children with viruses identified.

^b^Significant post-test pairwise comparison (P < 0.05) between malarial and non-malarial infection in children with bacteria identified.

^c^Significant post-test pairwise comparison (P < 0.05) between malarial and non-malarial infection in children without virus or bacterial nucleic acids identified.

^d^Significant post-test pairwise comparison (P < 0.05) between malarial-viral co-infection and no pathogen identified.

Any impairment of consciousness was associated with the presence of malaria on blood slide (χ^2^ = 39.6, *P* <0.001) and a history of multiple convulsions (χ^2^ = 9.8, *P* = 0.002). The presence of viral or bacterial NA was not associated with impaired consciousness.

Twenty-eight children (10%) had an adverse outcome, including 14 (5%) who died. The remaining 14 (5%) were discharged with neurological disabilities that included children with motor (n = 6), vision (n = 4), speech (n = 3) and sensory (n = 1) deficits. In multivariate analysis, an adverse outcome was independently associated with an abnormal level of consciousness (OR [CI_95_] 3.5 [1.4-8.8]), raised CSF protein (5.4 [1.8-14.9]) and age (1.014 per month [CI_95_ 1.002-1.026]). By contrast, malaria was associated with a lower chance of adverse outcome (0.30 [0.09-0.83]). After adjusting for these factors, the presence of viral or bacterial NA was not associated with an adverse outcome.

### Etiology and clinical patterns of disease in children with viral infections

#### i) Human herpes viruses

Human herpes viruses (HHV-6, HHV-7, HSV-1 and CMV) accounted for 91% of all viruses as single- and co-infections. Evidence of HHV-6 and HHV-7 infection in CSF was found in 21 (7%) of all children (Table 2). When compared to CMV, children with HHV-6 and −7 were more likely to have impaired consciousness (11 vs 3, *P* = 0.04), with trends towards higher proportions of complex seizures and adverse outcomes (Table 2). Four children with HHV-6 or −7 viruses had adverse outcomes, including one child with HHV-7 infection who died.

**Table 2:** **Clinical and laboratory features in patients with HHV-6 and −7 compared to those with CMV**

		HHV6, 7 (n = 21*)	CMV (n = 17* )	***P*** -value ^¶^
**Age (months)**		24 (8–56)	29 (6–38)	0.698
**Impaired consciousness**		11 (24)	3 (12)	0.04
**Complex seizure**		4 (19)	0 (0)	0.113
**Cerebrospinal fluid**	≥5 (cells/μL)	11 (52)	9 (53)	0.615
	Protein (≥1 g/L)	4 (19)	1 (6)	0.243
**Adverse outcome**	Permanent disability	3 (14)	1 (6)	0.355
	Mortality	1 (5)	0 (0)	

Data are numbers (percent) for dichotomous variables or medians (interquartile range) for continuous variables.

*The child with HHV-7/CMV coinfection is included in both groups; ^¶^Fisher’s exact test or Mann Whitney U test used.

The fatal HHV-7 case was a child aged 9 years admitted with multiple generalized convulsions. He was deeply comatose and had signs of meningism. He had a CSF lymphocyte count of 5 cells/μL, an elevated CSF protein of 3.0 g/L, and reduced CSF glucose (<5.5 mmol/L). Malaria microscopy as well as blood and CSF cultures were negative, and hemoglobin (111 g/L), total white blood cell density (15 × 10^9^/L), platelet density (368 × 10^9^/L), blood glucose (7.0 mmol/L) and blood lactate (1.0 mmol/L) were normal or within acceptable limits. Despite supportive therapy, his condition progressively deteriorated and he died after 3 days. Promptly-obtained post-mortem brain tissue showed extensive necrosis with neutrophilic infiltration, and there were reactive endothelial cells within cerebral vessels. Special histopathological stains for bacteria, fungi and *Mycobacterium* were negative. Cerebrospinal fluid PCR was subsequently positive for HHV-7 but negative for SP, Hib, *N. meningitidis*, picornaviruses, influenza viruses and flaviviruses. Nested PCR of brain tissue for malaria was also negative.

Seventeen children had evidence of CMV identified from CSF, 13 as single infections and four as mixed infections. Eleven (64%) of the children with CMV infection also had confirmed malaria at the time of admission. Only one child with CMV infection was discharged with cognitive impairment and none died.

Three children had evidence of HSV-1 infection, of which two had multiple co-infections (Figure 1). One child presenting with a focal, prolonged seizure lasting more than 15 minutes had HSV-1/HHV-7/SP identified in CSF as well *P. falciparum* in peripheral blood. Cerebrospinal fluid analysis revealed predominantly polymorphic white cells but there was no growth on bacterial culture. Another child had HSV-1/HHV-6/SP co-infection and presented with lower respiratory tract infection (LRTI) without convulsions and had a normal level of consciousness. The third child with HSV-1 mono-infection presented following a simple febrile convulsion. All three children were treated with intramuscular artemether and chloramphenicol and survived without neurological sequelae.

#### ii) Enterovirus

The only picornavirus identified in this study was an enterovirus identified from the CSF of an 8-month old infant who presented with respiratory symptoms and a single seizure lasting approximately 10 minutes. On admission, she was irritable. Lumbar puncture revealed normal CSF findings and she was discharged well. The enterovirus could not be further identified due to an insufficient volume of CSF.

#### iii) Flavivirus

Three children that had dengue type-2 infection presented to hospital within 9 days of each other. Two of these children were admitted on the same day with febrile convulsions and both were treated empirically with intramuscular artemether and chloramphenicol. One child had SP*/*dengue co-infection by nPCR (Figure 1) and another had *P. falciparum*/dengue co-infection. Both were discharged well without disability. The third child presented in deep coma and with clinical signs of meningeal irritation. He had a CSF cell count of 30 cells/μL (predominantly lymphocytes), but CSF and blood cultures were negative. He was also hypoglycemic (blood glucose 1.6 mmol/L), acidotic (blood lactate 15.1 mmol/L) and moderately thrombocytopenic (platelets 70 × 10^9^/L). He was diagnosed with viral encephalitis, and despite supportive therapy, he died within an hour of admission. His guardians declined a post mortem examination but CSF nPCR and plasma NS1 antigen tests were positive for dengue virus.

#### iv) Adenovirus

Adenovirus was not identified in any child with febrile encephalopathy.

## Discussion

The present study provides the first comprehensive data relating to the etiology of possible CNS infections in hospitalized children from a malaria-endemic area of PNG. Of 300 children without SSPE or proven bacterial/fungal meningitis, a possible candidate bacterial, viral or parasitic agent, or combination of agents, was identified in only 41%. There were few characteristics that differed between the six diagnostic groups, and thus no reliable clinical differentiation between the effects of bacteria or viruses in the CSF. Many children had more than one possible infection with overlap across different diagnostic groups.

Malaria appeared to have the most important effect on clinical characteristics of children with possible CNS infections. In multivariate analyses, the presence of malaria on blood slide was associated with the detection of bacterial or viral NA. Malaria was also independently associated with impaired consciousness and multiple convulsions, but after adjustment for other factors was inversely associated with an adverse outcome. Neither the presence of viral or bacterial DNA was independently associated with an adverse outcome or impaired consciousness. In post-hoc pairwise comparisons, children with malaria were older than their counterparts without malaria, regardless of the presence of viral or bacterial NA in the CSF.

Human herpes viruses accounted for the majority of the viral infections but there were some clinical differences within this group of related viruses. Children with HHV-6 or HHV-7 were much younger and more likely to have impaired consciousness than those with CMV infections, with an associated trend to a higher rate of complex seizures and adverse outcomes. These observations are consistent with the notion that acute infections associated with HHV6 and −7 might be more likely to present in very young children. The death of the child in whom HHV-7 was the only identifiable etiologic agent highlights the possibility that very severe disease and death may occasionally result from infections with these viruses.

Although HHV-6 and −7 have been shown to cause encephalitis and are associated with febrile convulsions in very young children [16]-[20], demonstrating a causal relationship between the presence of viral NA and clinical disease can be difficult. Like most HHVs, HHV-6 and −7 also are considered neurotropic [19]-[21] with the capacity to establish a latent reservoir. Re-activation of latent infections can cause clinical disease in immune-suppressed individuals [3],[22],[23], but data from developing countries suggest that malaria infection may also predispose to re-activation of HHVs. A small study of 49 Kenyan children with cerebral malaria found that HSV-1 was present in CSF from four children [5]. In a study from Uganda, there was an increase in Epstein-Barr virus in plasma and saliva from children with uncomplicated malaria which cleared with antimalarial treatment [3], suggesting that acute *P. falciparum* infection may cause HHV reactivation. Our data also support the hypothesis that malaria might cause re-activation of HHVs in some children but without major clinical consequences. Malaria was associated with a 2-fold increase in the chance of detecting a virus when compared to children without malaria. However, although HHVs made up >90% of possible viral pathogens isolated, they did not appear to have a characteristic clinical phenotype.

A recent Malawian study found that malaria-virus co-infections that included infections due to rabies, enteroviruses, mumps and adenoviruses were associated with an increased risk of seizures compared to either infection alone. Mortality in this group was also significantly higher than in children with malaria infection alone [2]. The apparent inconsistency between this finding and the results of the present study may reflect differences in the geographic distribution of encephalopathic viruses as well as their relative propensity to establish a latent reservoir. In addition, regional differences in malaria prevalence, incidence of severe malaria and associated mortality [5], temporal variability in enterovirus, adenovirus and/or arbovirus incidence [7], local zoonotic reservoirs contributing to the emergence of novel pathogens [8], and the choice of virus-specific primers that are included in the multiplex PCR tests employed during each study may all contribute to the distinctive local epidemiology of CNS infections.

Our data also confirm that viral pathogens including dengue and enterovirus that are commonly associated with clinically significant CNS infections occur, albeit rarely, in PNG children from the Madang area. Despite the low incidence of dengue in hospitalized children in the present study (1%), the child with dengue type-2 encephalitis died. This is the first reported case of death due to dengue encephalitis in PNG, highlighting that dengue should be considered an etiologic agent in PNG patients with undiagnosed CNS infections. Interestingly, the clustering of admissions of the three children with dengue type-2 infections indicates that transmission may be seasonal. Only one case of enterovirus infection was documented in our study, but was unable to be typed further.

In contrast to reports from developed [24],[25] and other tropical [2],[26],[27] countries, influenza, adenoviruses and other HHVs were not significant contributors to the burden of encephalopathy in our hospitalized children [7],[28]. Epstein-Barr virus, commonly associated with Burkitt’s lymphoma in malaria-endemic areas including PNG [29],[30], was not identified in the present study and neither was HHV-8, perhaps because PCR was performed on CSF rather than blood or saliva [3],[31].

The present study also raises the possibility of occult culture-negative bacterial infections in children with features of CNS infection. Bacterial infections not identified by blood or CSF culture, or by bacterial antigen testing, were identified in 6.7% of children using molecular diagnostic methods. Although one child with SP/Hib co-infection died, our data do not reveal any clear clinical pattern associated with occult bacterial infections. As with viral infections, the presence of malaria increased the chance of detecting a bacterial pathogen by approximately 2-fold. Although the clinical relevance of ‘incidental’ bacterial infections is not clear from our data, severely ill children with negative CSF or blood cultures might still benefit from empirical use of antibiotics.

Despite detailed investigations covering a large number of candidate etiologic agents, a large proportion of our children with possible CNS infection did not have a convincing final diagnosis. This warrants further studies in this group of vulnerable children, especially as increasingly sensitive molecular and other diagnostic tools continue to be developed [32]. In addition, non-infective causes of encephalopathy, such as environmental toxins, could also be considered where no pathogens are detected.

Our data have some limitations, particularly with the challenges of delineating between acute infections and reactivation of HHVs. Firstly, sophisticated molecular analyses differentiating HHV-6 DNA from actively replicating virus or forms associated with chromosomal integrated DNA were not performed [4]. Secondly, we did not perform tests to define whether children with HHV-6 had HHV-6A or 6B. Thirdly, we did not have neuroimaging diagnostics in our setting that could assist in the diagnosis and management of brain infections. Finally, we did not have plasma available for serological tests such as species-specific immunoglobulin M or G that might assist with making a diagnosis of an acute or latent infection, respectively.

## Conclusion

The present study indicates that viral infections such as dengue, enterovirus and perhaps HHV-6 and −7 can cause serious CNS disease in some PNG children. However, there is no apparent effect of the presence of viral NA on clinical features and outcome in this group. By contrast, malaria has a strong positive effect on the likelihood of detection of viral and bacterial NA, is itself associated with clinically important characteristics such as impaired consciousness, and is an independent predictor of a good outcome. This suggests that HHVs may be the result of reactivation from a latent reservoir as a result of malaria without clinical sequelae in most cases. Although the capacity to diagnose significant viral infections in PNG is limited, these data should inform clinicians as to differential diagnoses in an encephalopathic child that should now include dengue and enterovirus infections. Antiviral medications such as aciclovir are routinely not available in PNG. Although antiviral medications have been used successfully in a small number of immunocompetent patients with encephalomyelitis due to HHV-6 [33], the lack of data from well-designed studies, high medication costs and the lack of capacity to make a diagnosis, preclude the incorporation of antiviral recommendations into standard treatment guidelines for countries like PNG. The present data do not argue for a change to these guidelines.

## Authors’ original submitted files for images

Below are the links to the authors’ original submitted files for images. Authors’ original file for figure 1 Authors’ original file for figure 2
