# Supplementary figures and images for: Viral pathogens in children hospitalized with features of central nervous system infection in a malaria-endemic region of Papua New Guinea
Source: BMC Infect Dis. 2014 Nov 26;14:630. doi: 10.1186/s12879-014-0630-0 (PMC4260243; doi:10.1186/s12879-014-0630-0)

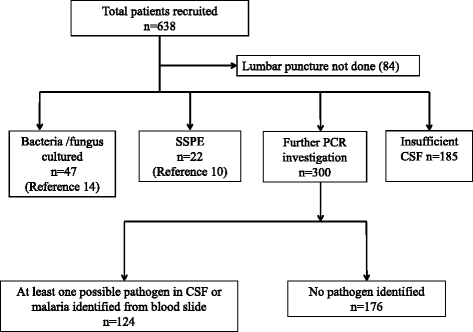

Supplement: Supplementary file 1 — Authors’ original file for figure 1 [file 12879_2014_630_MOESM1_ESM.gif]

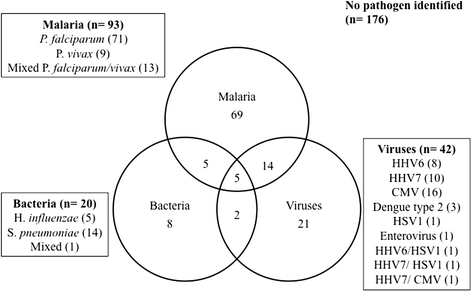

Supplement: Supplementary file 2 — Authors’ original file for figure 2 [file 12879_2014_630_MOESM2_ESM.gif]
